# Moxibustion prevents tripterygium glycoside-induced oligoasthenoteratozoospermia in rats via reduced oxidative stress and modulation of the Nrf2/HO-1 signaling pathway

**DOI:** 10.18632/aging.205475

**Published:** 2024-01-25

**Authors:** Shangjie Liang, Yaqun Yin, Zhizi Zhang, Yansu Fang, Ge Lu, Hongxiao Li, Yaoli Yin, Meihong Shen

**Affiliations:** 1 College of Acupuncture, Moxibustion and Tuina, Nanjing University of Chinese Medicine, Nanjing 210023, Jiangsu, China; 2 Key Laboratory of Acupuncture and Medicine Research of Ministry of Education, Nanjing University of Chinese Medicine, Nanjing 210023, Jiangsu, China

**Keywords:** moxibustion, oligoasthenoteratozoospermia, sperm quality, oxidative stress, Nrf2, HO-1

## Abstract

Oligoasthenoteratozoospermia (OAT) decreases male fertility, seriously affecting the production of offspring. This study clarified the preventive impact of different moxibustion frequencies on OAT and selected the optimal frequency to elucidate the underlying mechanism. An OAT rat model was constructed by gavage of tripterygium glycosides (TGS) suspension. Daily moxibustion (DM) or alternate-day moxibustion (ADM) was administered on the day of TGS suspension administration. Finally, we selected DM for further study based on sperm quality and DNA fragmentation index, testicular and epididymal morphology, and reproductive hormone level results. Subsequently, the oxidative stress (OS) status was evaluated by observing the OS indices levels; malondialdehyde (MDA), 8-hydroxy-deoxyguanosine (8-OHdG), total antioxidant capacity (T-AOC), and total superoxide dismutase (T-SOD) in testicular tissue using colorimetry and enzyme-linked immunosorbent assay. Furthermore, heme oxygenase 1 (HO-1) and nuclear factor erythropoietin-2-related factor 2 (Nrf2) were evaluated using Western blotting. Immunohistochemistry was employed to locate and assess the expression of HO-1 and Nrf2 protein, while quantitative real-time polymerase chain reaction was utilized to detect their mRNA expression. MDA and 8-OHdG levels decreased following DM treatment, while T-SOD and T-AOC increased, suggesting that DM may prevent TGS-induced OAT in rats by decreasing OS in the testis. Furthermore, protein and mRNA expression of Nrf2 and HO-1 in the testis were elevated, indicating that DM may reduce OS by activating the signaling pathway of Nrf2/HO-1. Therefore, DM could prevent OAT in rats via the Nrf2/HO-1 pathway, thereby presenting a promising therapeutic approach against OAT.

## INTRODUCTION

Oligoasthenoteratozoospermia (OAT), characterized by a significant decrease in sperm concentration and motility, accompanied by a considerable rise in sperm malformation rate, is a major cause of male infertility [1]. Some studies have attributed OAT to hypogonadism caused by insufficient testosterone secretion [2, 3]. Therefore, in clinical practice, testosterone replacement therapy is often administered empirically or experimentally for treatment [4, 5]. However, its application has limitations that hinder its widespread use [6]. It may also raise the cardiovascular disorder risk, and its use in clinical practice therefore remains controversial [7]. OAT may be directly linked to oxidative stress (OS) [8]. Previous clinical trials [9–11] have shown significant reactive oxygen species amounts in the semen of individuals with OAT, and animal experiments [12, 13] have shown significantly increased 8-hydroxy-deoxyguanosine (8-OHdG) and malondialdehyde (MDA) levels in the testis of OAT rats. This indicates that OS-mediated testicular injury may be the core mechanism underlying the occurrence of OAT. The nuclear factor erythropoietin-2-related factor 2 (Nrf2) plays a vital part in protecting the testis from OS by enhancing cellular antioxidant defense mechanisms [14]. Studies on Nrf2 knockout mice have reported decreased sperm concentration and motility, and significantly lower fertility rates, compared with control mice [15]. Activating Nrf2 expression and its downstream heme oxygenase 1 (HO-1) expression can downregulate OS and improve sperm quality [16]. Therefore, it can be postulated that activating the Nrf2/HO-1 signaling pathway and subsequent mitigation of OS is a potential mechanism in averting OAT.

Moxibustion is a conventional Chinese medicinal modality that involves the application of compressed powdered herbal material (moxa) for acupoints to generate warmth in the meridians. The efficacious components of mugwort are infiltrated into the acupoints by warm and near-infrared stimulation, which has preventive and therapeutic effects on diseases [17]. Recent clinical research has shown that moxibustion has a good curative effect on knee osteoarthritis [18], polycystic ovary syndrome [19], and ulcerative colitis [20]. The therapeutic effect of moxibustion is believed to be related to its intervention frequency [21, 22]; although most frequencies are effective, there are differences. It is therefore necessary to optimize moxibustion frequencies to intervention measures for practical application. In addition, previous studies [23, 24] have found that moxibustion has significant antioxidant effects and is connected with the Nrf2/HO-1 signaling pathway. Nevertheless, data on the moxibustion pathway for preventing and treating OAT remain limited.

**Figure 1:**
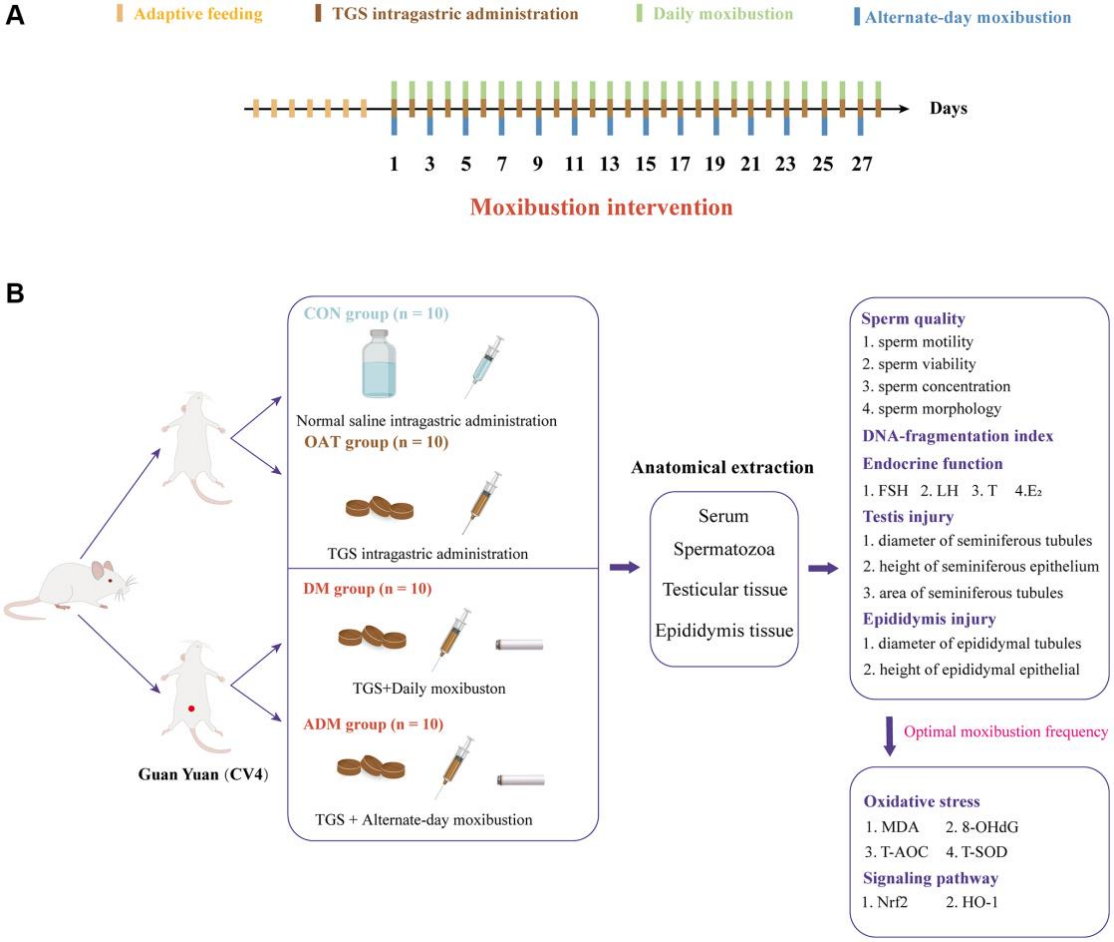
(**A**) Intervention time points in rats. (**B**) Experimental strategies and main indicators in rats.

Therefore, this study examined the different moxibustion frequencies’ impact on tripterygium glycosides (TGS)-induced OAT in rats and explored the potential underlying molecular mechanisms (Figure 1). The results suggested that Daily moxibustion (DM) prevents OAT, probably via decreasing OS and regulating the Nrf2/HO-1 signaling pathway. The findings of this study provide novel insights into the use of moxibustion in the treatment of OAT.

## RESULTS

### Moxibustion prevented the decline of sperm quality in rats with TGS-induced OAT

**Figure 2:**
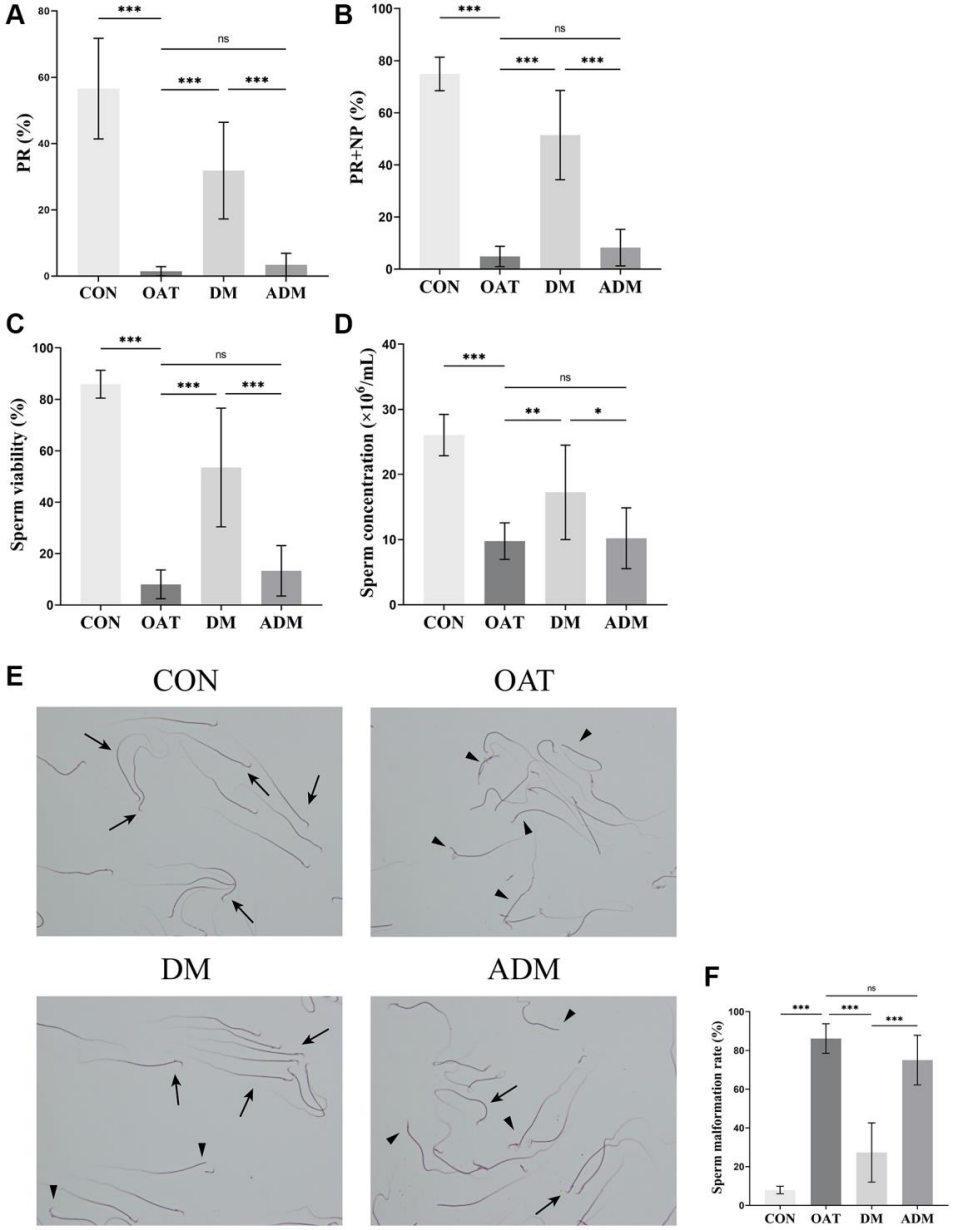
**Effects of moxibustion on the sperm quality of rats with TGS-induced OAT.** (**A**) PR. (**B**) PR+NP. (**C**) Sperm viability. (**D**) Sperm concentration. (**E**) Morphological changes of sperm observed using 2% eosin staining (×400). Black triangle: abnormal sperm; black arrow: normal sperm. (**F**) Sperm malformation rate. *n* = 10 in each group. ^*^P < 0.05, ^**^P < 0.01, ^***^P < 0.001; Abbreviation: ns: not significant.

Sperm quality assessment is the most important indicator of male fertility and is considered the gold standard in clinical practice [25]. As demonstrated in Figure 2, progressive motility (PR), sperm total motility (PR+NP), sperm viability, and sperm concentration in the model group significantly reduced (*P* < 0.001 for all; Figure 2A–2D), while the deformity rate increased (*P* < 0.001; Figure 2E, 2F), indicating that the OAT model was successfully established. After DM, sperm quality including PR, PR+NP, sperm viability, sperm concentration increased and sperm malformation rate decreased significantly (*P* < 0.001, *P* < 0.001, *P* < 0.001, *P* < 0.01, and *P* < 0.001, respectively). However, there were no significant improvements in the alternate-day moxibustion (ADM) group compared with the OAT group (*P* > 0.05, for all). In contrast with the DM group, the PR, PR+NP, sperm viability and sperm concentration of the ADM group decreased (*P* < 0.001, *P* < 0.001, *P* < 0.001 and *P* < 0.05, respectively), and there was an increase of the sperm malformation rate (*P* < 0.001).

### Moxibustion preserved sperm DNA integrity in rats with TGS-induced OAT

**Figure 3:**
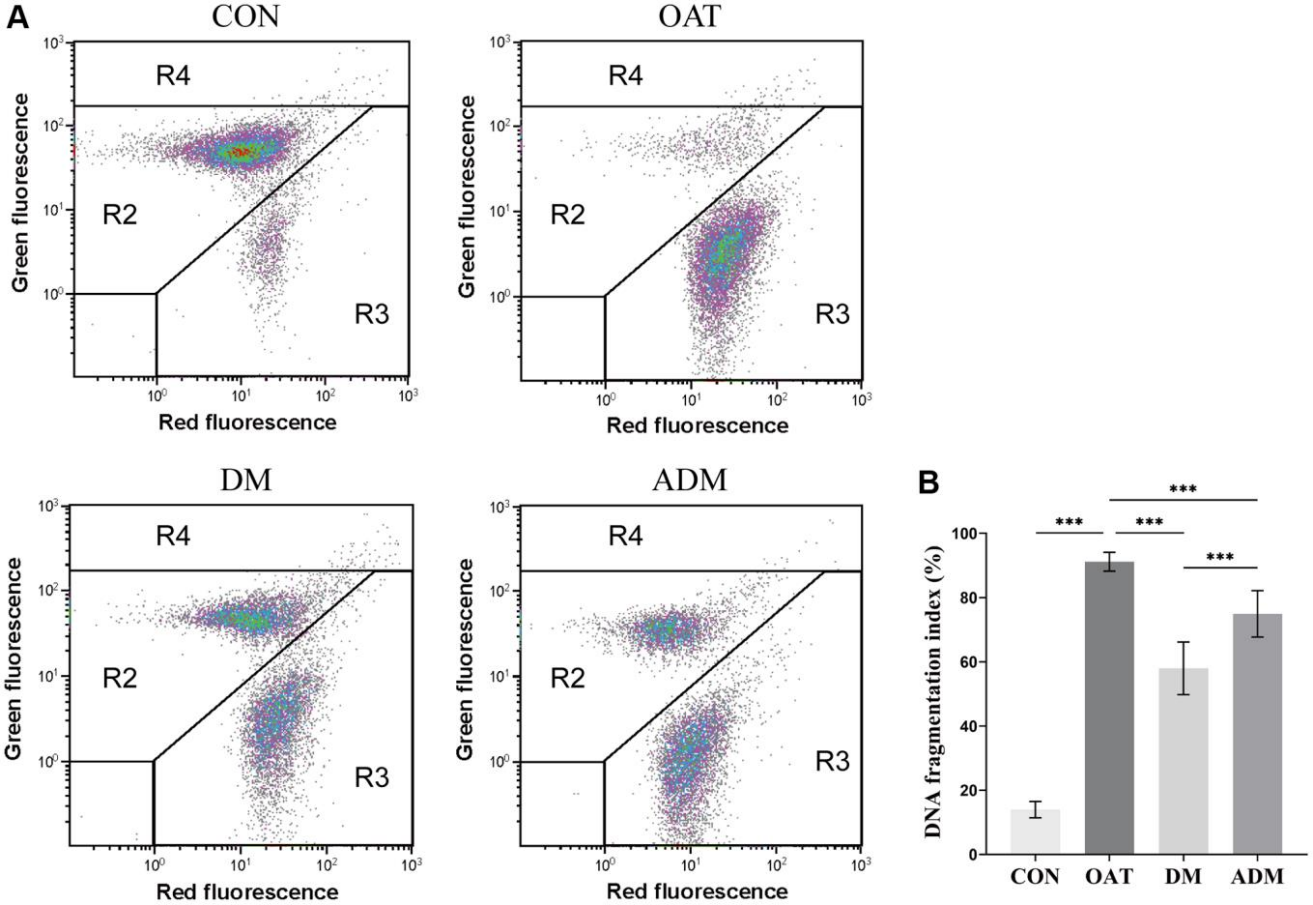
**Effects of moxibustion on rat sperm DNA integrity detected using flow cytometry.** (**A**) Representative images of CON, OAT, DM, and ADM groups. Each dot represents a spermatozoon characterized by the amount of double-stranded DNA (green fluorescence) or single-stranded DNA (red fluorescence). R2 = normal sperm population. R3 = DNA denatured sperm population. R4 = immature sperm population. (**B**) DNA fragmentation index (*n* = 6). ^***^P < 0.001.

The sperm chromatin structure of each group of rats was examined through sperm chromatin structure assay using sperm cells from the caudal epididymis (Figure 3A, 3B). The study compared the sperm DNA fragmentation, as evaluated by DNA fragmentation index (DFI), across various groups.

The results indicated a significant elevation in DFI among the OAT group when compared with Control (CON) group (*P* < 0.001). Both moxibustion groups showed reduced sperm DFI (*P* < 0.001 for both), whereas the DM group exhibited a reduced DFI compared with the ADM group (*P* < 0.001).

### Moxibustion prevented testicular damage in rats with TGS-induced OAT

**Figure 4:**
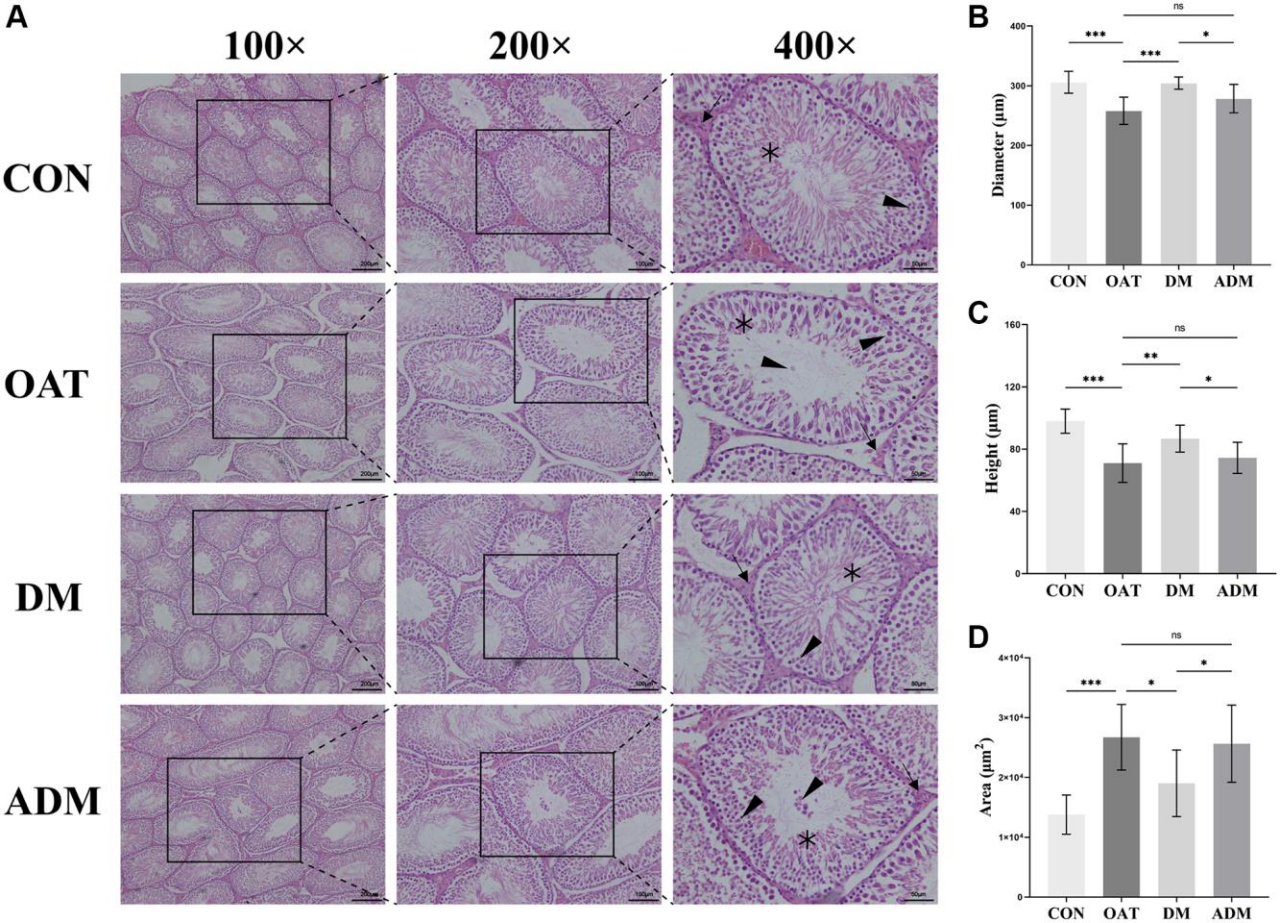
**Moxibustion prevents testicular damage in rats with TGS-induced OAT.** (**A**) Representative images of testicular histology detected by H&E staining (×100; ×200; ×400). Black triangle: spermatogenic cells; black arrow: Leydig cells; black asterisk: Sertoli cells. (**B**) Diameter of seminiferous tubules. (**C**) Height of the seminiferous epithelium. (**D**) Area of seminiferous tubules. *n* = 10 in each group. ^*^P < 0.05, ^**^P < 0.01, ^***^P < 0.001; Abbreviation: ns: not significant.

The results depicted in Figure 4A indicated that the seminiferous tubules of the control rats exhibited moderately circular or oval outlines, and a stratified seminiferous epithelium with cells of the spermatogenic series was observed. Pathological manifestations in the OAT group included waste of spermatogenic cells, sloughed spermatogenic cells, a lessening in the number of spermatogenic cells, seminiferous tubules damage, cytoplasmic vacuolization of Sertoli cells, and atrophy of Leydig cells. The testicular tissue of OAT rats was significantly improved after moxibustion intervention, reflected by the increase in Sertoli and Leydig cells, and the spermatogenic cells were neatly arranged. In contrast with the ADM group, the improvement was more pronounced in the DM group.

In addition, the extent of the seminiferous epithelium, the diameter of the seminiferous tubule, and the seminiferous tubule area were assessed to quantify the alterations in testicular histology. Compared with the CON group, the seminiferous tubule diameter and the seminiferous epithelium height in rats from the OAT group were significantly reduced (*P* < 0.001 for both; Figure 4B, 4C), and the area of seminiferous tubules was larger (*P* < 0.001; Figure 4D). In comparison with the OAT group, the seminiferous tubule diameter and the height of the seminiferous epithelium in rats from the DM group were significantly increased (*P* < 0.001 and *P* < 0.01, respectively; Figure 4B, 4C), and the seminiferous tubules area decreased (*P* < 0.05; Figure 4D). However, the ADM group did not exhibit a statistically significant enhancement in seminiferous tubules (*P* > 0.05; Figure 4B–4D).

### Moxibustion prevented epididymal damage in rats with TGS-induced OAT

**Figure 5:**
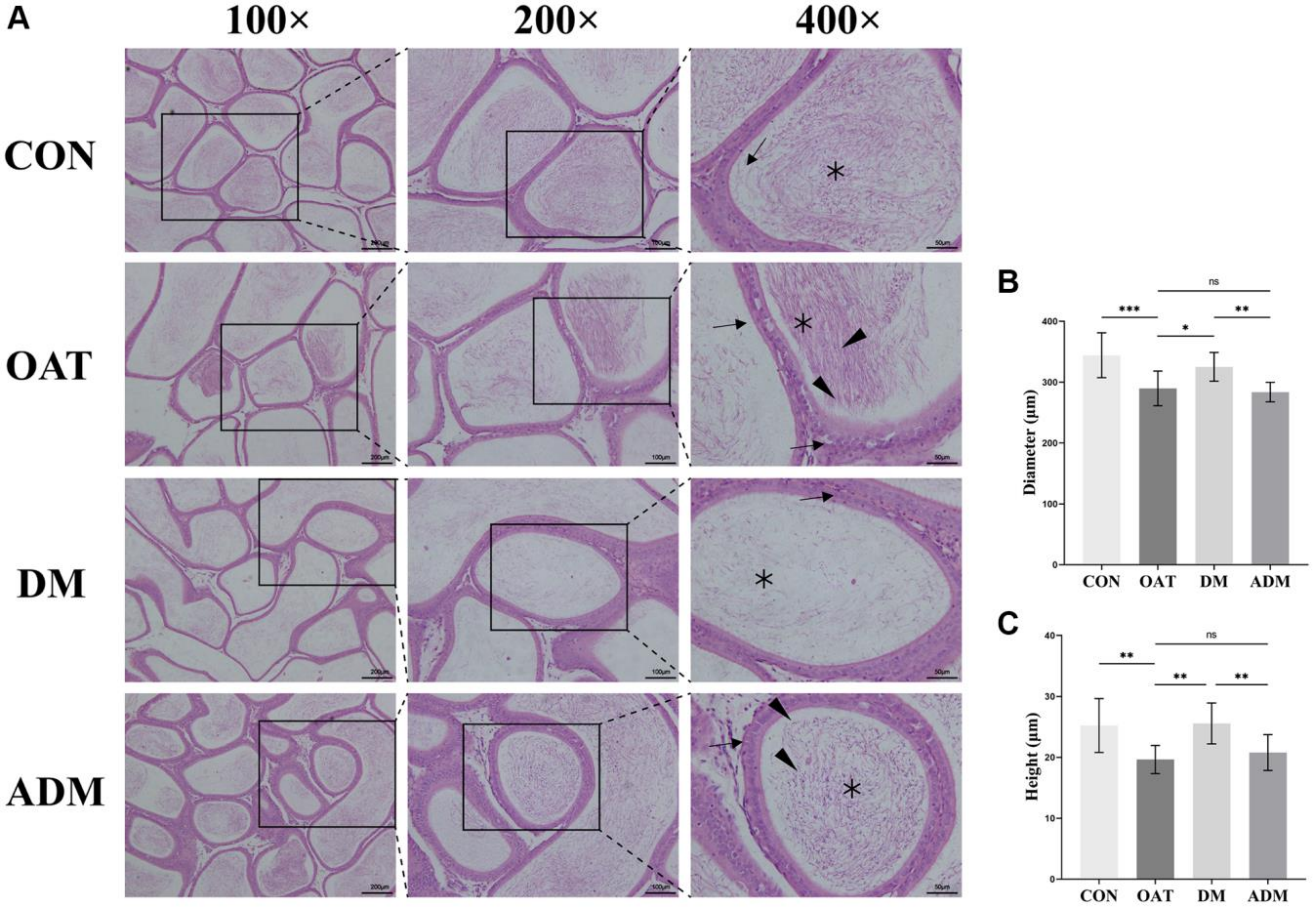
**Moxibustion has a protective effect on preventing epididymal damage in rats with TGS-induced OAT.** (**A**) Representative images of epididymal histology detected using H&E staining (×100; ×200; ×400). Black triangle: spermatogenic cells; black arrow: epididymal epithelium cells; black asterisk: spermatozoa. (**B**) Diameter of the epididymal duct; (**C**) Height of the epididymal epithelium. *n* = 10 in each group. ^*^P < 0.05, ^**^P < 0.01, ^***^P < 0.001; Abbreviation: ns: not significant.

As shown in Figure 5A, control rats showed normal epididymal histological architecture with numerous mature spermatozoa in the epididymal lumen, and the epididymal epithelium cells were orderly and neatly arranged. Rats in the OAT group showed ductus epididymis impairment with a large number of shed epididymal epithelium cells, and spermatogenic cells dropping from seminiferous tubules were present in the lumen of the epididymal duct. The epididymis tissue of the DM group was significantly improved, with reduced shedding of epididymal epithelial cells and neatly arranged epididymal epithelial cells. Although there was a certain degree of improvement in the ADM group, there remained some deficiencies compared with the DM group. To compare the differences among groups, we also measured the tubular diameters and epithelial height of the epididymis to quantify the change in epididymal morphology (Figure 5B, 5C). In contrast with the CON group, the tubular diameters and epithelial height of the epididymis in the group OAT were significantly reduced (*P* < 0.001 and *P* < 0.01, respectively). The epididymis tubular diameters and epithelial height were all significantly elevated in the DM group (*P* < 0.05 and *P* < 0.01, respectively). The ADM group did not yield a statistically significant enhancement in the epididymal duct (*P* > 0.05).

### Moxibustion regulated hormonal imbalance in rats with TGS-induced OAT

**Figure 6:**
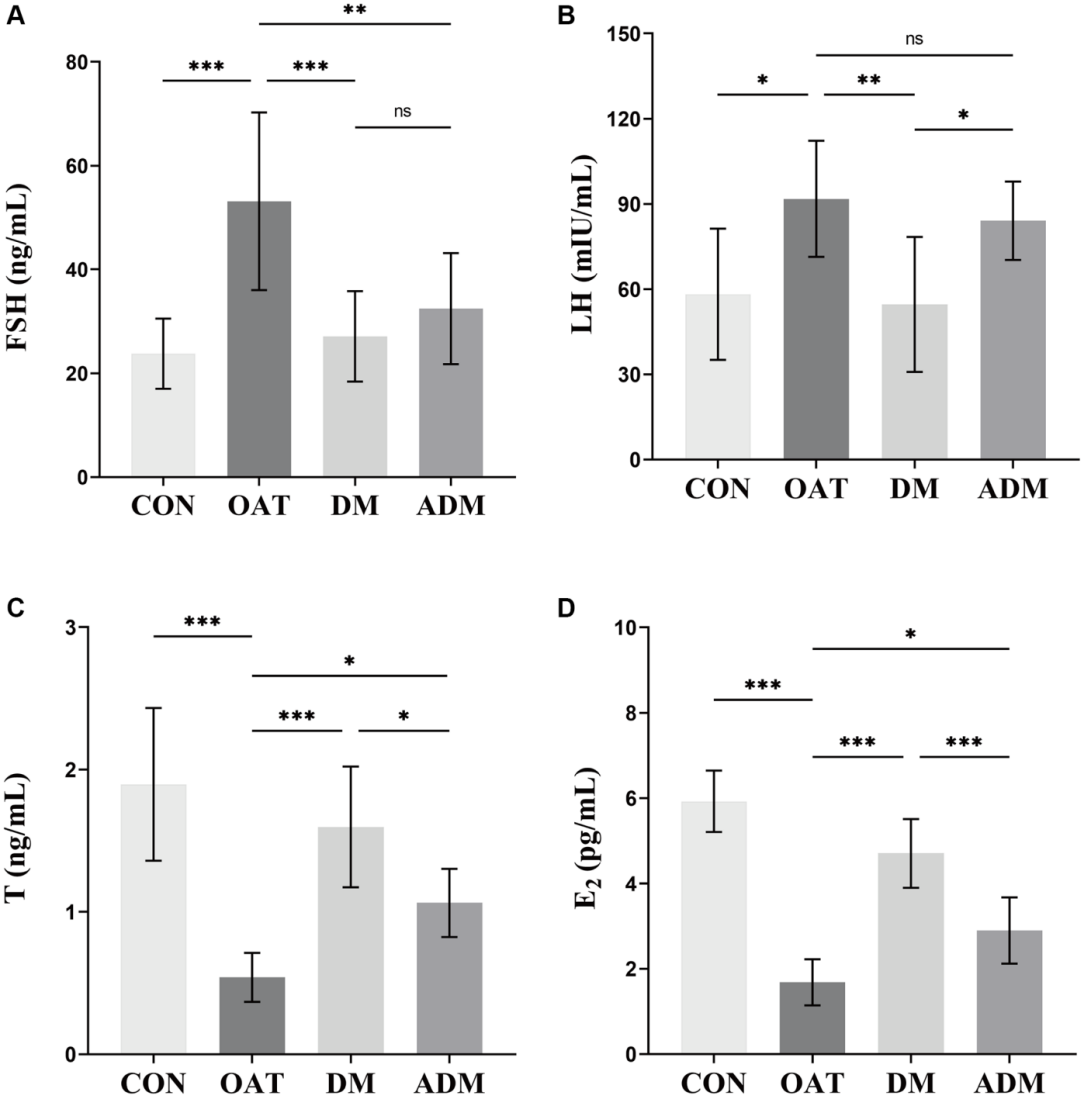
**Effects of moxibustion on serum hormone levels in rats with TGS-induced OAT.** (**A**) FSH. (**B**) LH. (**C**) T. (**D**) E_2_. *n* = 8 in each group. ^*^P < 0.05, ^**^P < 0.01, ^***^P < 0.001; Abbreviation: ns: not significant.

The regulation of testicular roles is primarily controlled by the neuroendocrine hypothalamo-hypophysial-gonadal axis feedback system, which is contingent upon the serum level of reproductive hormones; therefore, a decline in testicular function is directly reflected in hormone level disturbances [26]. As illustrated in Figure 6, the OAT group exhibited a significant rise in follicle-stimulating hormone (FSH) and luteinizing hormone (LH) serum levels in contrast to the CON group (*P* < 0.001 and *P* < 0.05, respectively, Figure 6A, 6B), while testosterone (T) and estradiol (E_2_) levels were decreased (*P* < 0.001 for both, Figure 6C, 6D). In contrast with the OAT group, the disturbed hormone levels were balanced in the DM group, as shown by the reduced serum levels of LH and FSH (*P* < 0.01 and *P* < 0.001, respectively) and increased levels of T and E_2_ (*P* < 0.001 for both). The FSH level also decreased (*P* < 0.01), while that of T and E_2_ increased (*P* < 0.05 and *P* < 0.05, respectively) in the ADM group. The DM group exhibited a better ability to regulate hormones than the ADM group.

### DM decreased testicular tissue oxidative damage in rats with TGS-induced OAT

Based on the above outcomes, the overall impact of DM on OAT was better than that of ADM. To investigate the potential mechanism of moxibustion in preventing OAT, DM was selected for further exploration to reveal its relationship with OS.

**Figure 7:**
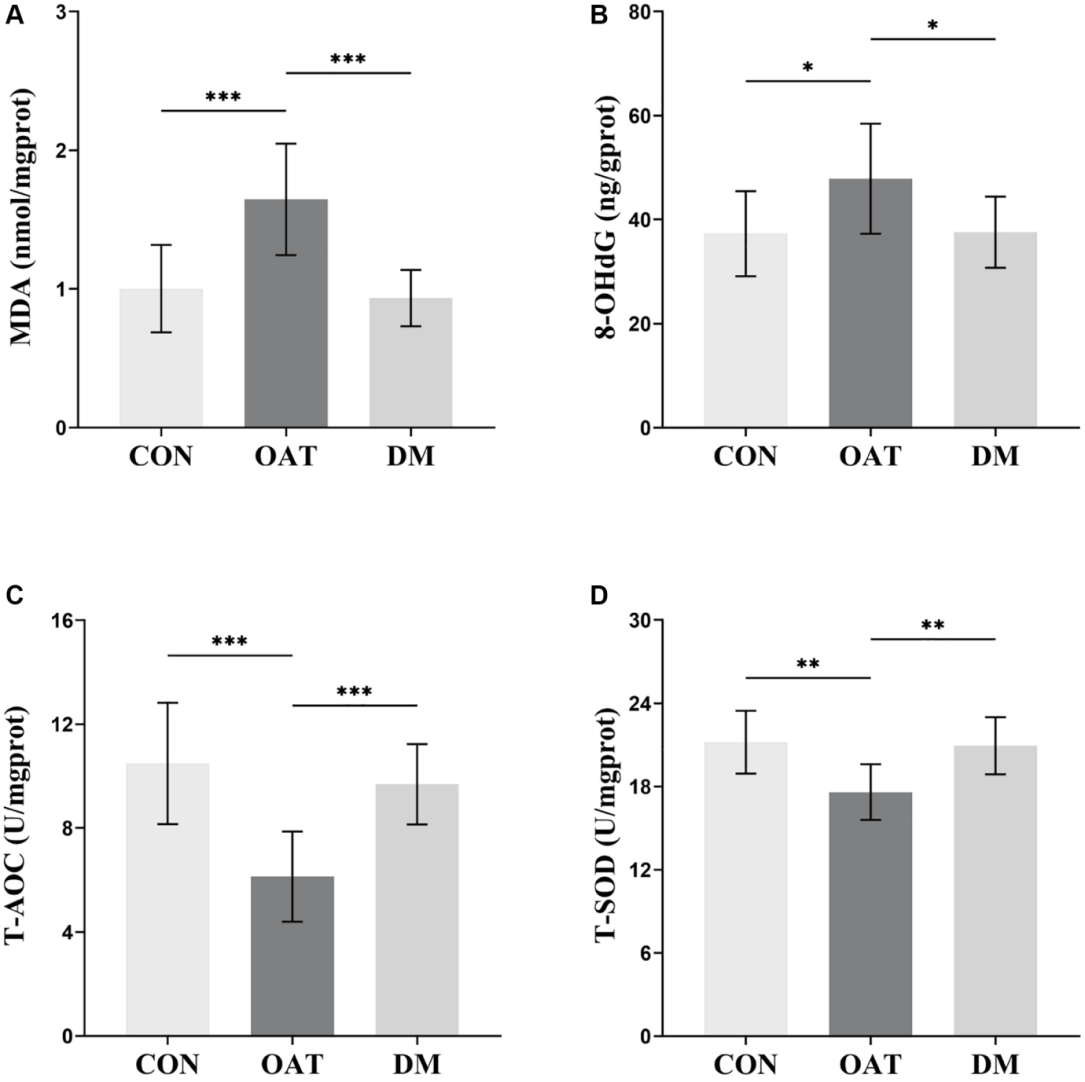
**Effects of DM on testicular tissue OS in rats with TGS-induced OAT.** (**A**) MDA. (**B**) 8-OHdG. (**C**) T-AOC. (**D**) T-SOD. *n* = 10 in each group; ^*^P < 0.05, ^**^P < 0.01, ^***^P < 0.001.

As presented in Figure 7, the testicular tissues of OAT group rats exhibited significantly high levels of MDA and 8-OHdG (*P* < 0.001 and *P* < 0.05, respectively, Figure 7A, 7B), but low levels of total antioxidant capacity (T-AOC) and total superoxide dismutase (T-SOD) (*P* < 0.001 and *P* < 0.01, respectively, Figure 7C, 7D) in contrast with the CON group. MDA and 8-OHdG levels in the testicular tissues of the DM group decreased (*P* < 0.001 and *P* < 0.05, respectively, Figure 7A, 7B), while T-AOC and T-SOD levels increased (*P* < 0.001 and *P* < 0.01, respectively, Figure 7C, 7D) compared with the OAT group.

### DM activated the Nrf2/HO-1 signaling pathway in rats with TGS-induced OAT

**Figure 8:**
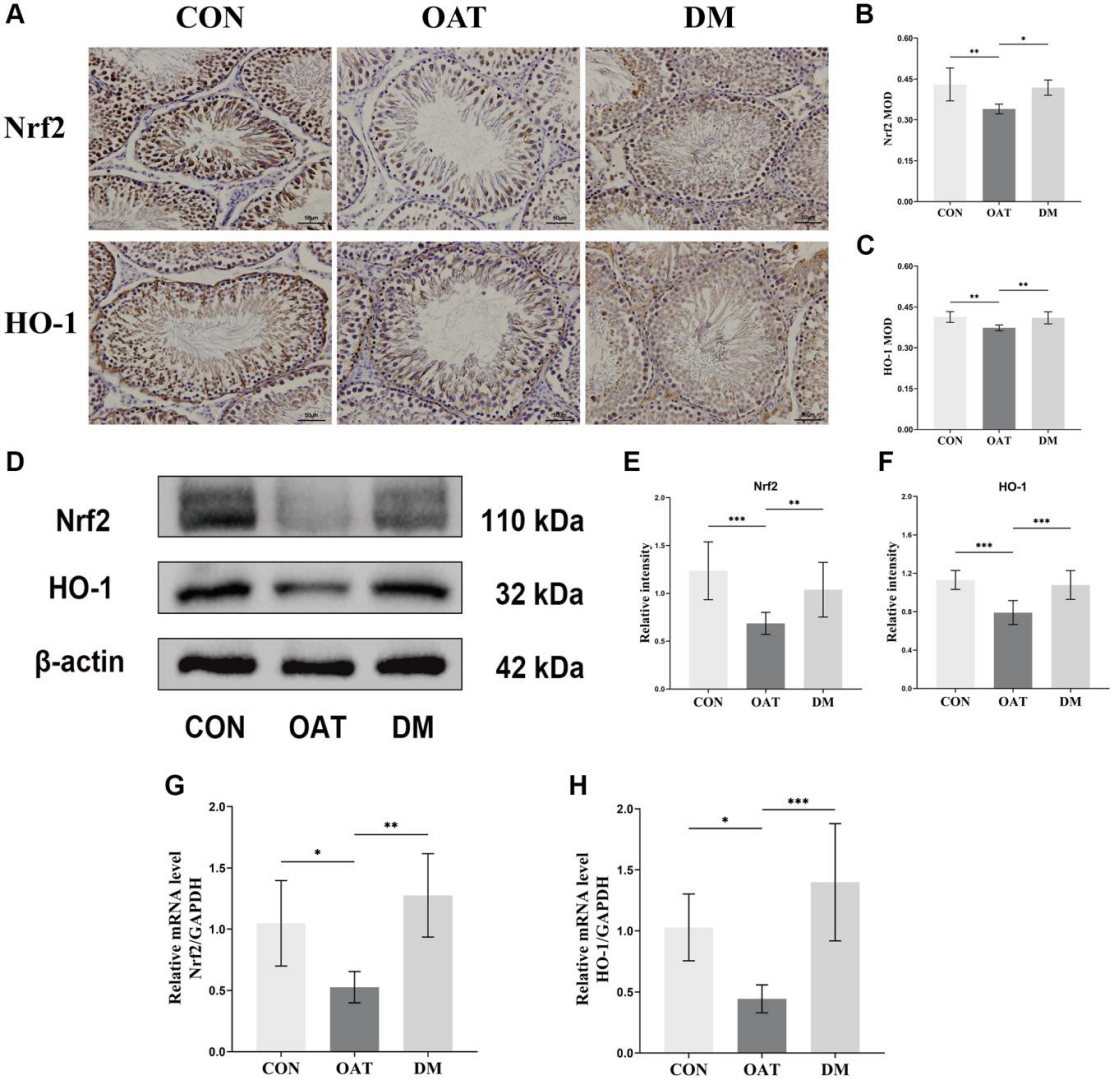
**Regulatory effect of DM on the Nrf2/HO-1 signaling pathway in rats with TGS-induced OAT.** (**A**) IHC staining of Nrf2 and HO-1 in testicular tissues (×400; scale bar: 50 μm). (**B**, **C**) MOD values of Nfr2 and HO-1 (*n* = 6). (**D**) Western blotting analysis of the indicated proteins in each group. (**E**, **F**) Quantitative analysis of Nfr2 and HO-1 expression from Western blots (*n* = 3). (**G**, **H**) mRNA expression of Nrf2 and HO-1 in testicular tissues (*n* = 6). ^*^P < 0.05, ^**^P < 0.01, ^***^P < 0.001.

We examined whether the Nrf2/HO-1 signaling pathway was associated with the reduction of testicular OS by DM in OAT rats. The immunohistochemistry (IHC) of the testicular tissue revealed that Nrf2 and HO-1 were primarily expressed in Sertoli and spermatogenic cells (Figure 8A). Quantitative results were expressed as the MOD (Figure 8B, 8C); the OAT group had significantly lower HO-1 and Nrf2 expressions than the CON group (*P* < 0.01 for both), whereas those of the DM group were greater than those of the OAT group (*P* < 0.01, *P* < 0.05, respectively). The levels of Nrf2 and HO-1 protein expression detected by Western blotting are illustrated in Figure 8D. There were elevated Nrf2 and HO-1 protein expression levels in the DM group in contrast with the OAT group (*P* < 0.01 and *P* < 0.001, respectively; Figure 8E, 8F). The levels of Nrf2 and HO-1 mRNA expression detected using quantitative real-time PCR (qRT-PCR) are shown in Figure 8G, 8H. In contrast with the OAT group, the expression of Nrf2 and HO-1 mRNA was higher in the DM group (*P* < 0.01 and *P* < 0.001, respectively).

These outcomes showed that the trend of DM increasing the Nrf2 and HO-1 was consistent in both the IHC quantitative analysis and in the expression levels of protein and transcription, indicating that DM may prevent TGS-induced OAT in rats by activating the signaling pathway of Nrf2/HO-1.

## DISCUSSION

As a complex male reproductive disease, OAT is a major cause of male infertility and has received widespread attention worldwide [27]. Due to its unclear pathogenesis, clinical treatment for OAT is limited. Therefore, developing more effective methods for the prevention and management of OAT is necessary. Moxibustion has been utilized for the treatment of OAT for centuries, and clinical and experimental studies have confirmed its effectiveness [28, 29]. Guanyuan (CV4) can nourish the kidney and is often used to treat diseases related to male infertility. CV4 can effectively treat male infertility and improve sperm quality [30], exert antioxidant stress effects, and regulate sex hormone levels [31, 32]. Although it is widely used in clinical practice, there are only a few reports delineating its underlying mechanisms in alleviating OAT. Relevant studies [21, 33] suggested that the effect of moxibustion on OAT depends on its intervention frequency, with different frequencies producing varying effects. In this study, we constructed an OAT rat model to evaluate the effects of different moxibustion frequencies on the disease and selected the optimal moxibustion frequency based on the results for further research of its mechanism.

Sperm qualities, such as motility, viability, concentration, and malformation rate, determine the ability of male reproductive function [34]. The sperm DFI determines the fertilization ability of sperm, formation of prokaryotes, embryo implantation after fertilization, and offspring health. Semen routine analysis parameters cannot detect subtle DNA defects in sperm sterility or assess the integrity of sperm chromatin. Therefore, DFI is an independent indicator of semen quality [35]. Rats in the OAT group exhibited a significant decline in sperm quality in contrast with the CON group, consistent with the basic features of OAT [36]. Noteworthy, DM prevented the deterioration in sperm quality, while ADM was not very effective. However, both frequencies reduced sperm DFI.

The optimum spermatogenic function of the testis requires normal sex hormone levels. FSH and LH secreted by the pituitary gland act on the Leydig and Sertoli cells within the testis, increasing the secretion of T and E_2_ and promoting the differentiation of spermatogenic cells. The level of serum sex hormones reflects the spermatogenic function of the testis. Studies have shown that the sex hormone levels in infertile men present a disordered state [37, 38]. In this study, the OAT group showed a significant reduction and shrinkage of Sertoli and Leydig cells in the testis compared with the CON group, which impaired the production of T and E_2_ by interfering with the action of LH on Leydig cells and reducing the concentration of T. Moreover, the binding ability of FSH to its receptor on Sertoli cells was diminished, leading to a marked elevation of FSH. Notable, both DM and ADM could effectively decrease FSH levels and increase T and E_2_ levels compared with the OAT group, suggesting that moxibustion may balance sex hormone levels and facilitate normal spermatogenesis by protecting Sertoli and Leydig cells from damage in testicular tissue. However, ADM was less effective than DM at balancing sex hormone levels and did not significantly improve levels of LH. This indicates that moxibustion has various effects according to the frequency of its application.

The moxibustion effect is initiated by stimulating the temperature of the superficial or deep tissue of the acupoint skin, activating the local specific receptors, heat-sensitive immune cells, and heat shock proteins in the acupoint, inducing various local effects. Through neural and humoral pathways, the moxibustion thermal stimulation signal and subsequent effects are transmitted to distant organs and the whole body, inducing subsequent effects in specific target organs and systems in the distant areas, achieving therapeutic outcomes [39]. Our results indicated that moxibustion could effectively prevent OAT by improving sperm quality, reducing sperm DFI, balancing hormone levels, and restoring damage to the testes and epididymis. Notably, different frequencies of moxibustion have different effects. Previous studies have shown that the efficacy of moxibustion is influenced by frequency [40, 41]. Although, in this study, some hormone indicators in the ADM group were changed, the improvement in sperm quality was not significant, possibly due to insufficient moxibustion frequency. This indicates that the moxibustion impact is directly connected to its frequency. To further elucidate the mechanism of action of moxibustion in preventing OAT, we selected DM for further research.

OS is the main pathogenesis of OAT [42]. In clinical practice, semen from patients with OAT has been shown to have significantly high levels of reactive oxygen species and MDA, which induces OS leading to considerable sperm mortality [43, 44]. After using antioxidants, the T level of male patients with infertility considerably increased and the sperm quality considerably improved [43, 44]. This has also been shown in experimental studies. Indeed, when using ornidazole or a high-fat diet to induce OS damage in the testes of mice/rats, hormone levels were perturbed and sperm quality decreased [45–47]. Based on current research findings, moxibustion is believed to have substantial antioxidant effects. A clinical study has shown that moxibustion can effectively reduce MDA and increase SOD in the seminal plasma of patients with OAT, reduce OS levels, and improve sperm quality [48]. Furthermore, moxibustion can increase antioxidant stress capacity by increasing the levels of SOD and HO-1 [24]. Although the antioxidant effects of moxibustion have been confirmed in multiple reports, research on the mechanisms of moxibustion in the prevention and treatment of OAT remains limited. In this study, in contrast with the CON group, rats in the OAT group exhibited a substantial rise in the OS products MDA and 8-OHdG in testicular tissue. This indicated that oxidative damage occurred in the cell membrane of spermatogenic cells in testicular tissue, and this oxidative damage caused DNA damage within cells, which was also reflected in increased sperm DFI [49]. In addition, a notable reduction in the levels of T-AOC and T-SOD antioxidant factors was observed in the testicular tissue, suggesting that excessive OS in the testis may inhibit the endogenous antioxidant system. Compared with the OAT group, the DM group showed a significant decrease in MDA and 8-OHdG content and an increase in T-AOC and T-SOD content. Consistent with a previous study [50], these findings demonstrate that DM has a good antioxidative stress effect.

This study showed that the effect of DM in preventing OAT was associated with the alleviation of testicular OS, although the underlying mechanism remains unclear. Previous studies [35, 36] have shown that the Nrf2/HO-1 signaling pathway is strictly connected to OS. Nrf2 is involved in maintaining intracellular redox balance to prevent OS [51]. After OS, Nrf2 activation can directly induce HO-1 production. Activating HO-1 could effectively reduce OS indicators such as MDA in the testis, while increasing antioxidant indicators such as SOD, effectively resisting OS [52]. The Nrf2/HO-1 pathway has been identified as a possible treatment option for OAT [53]. We observed that the transcription and protein levels of Nrf2 and HO-1 were considerably downregulated in the OAT group, suggesting that the cause of OS is the Nrf2/HO-1 signaling pathway suppression. Following DM intervention the transcription and protein levels of Nrf2 and HO-1 were significantly elevated compared with the OAT group, consistent with previous findings [23, 50, 54], suggesting that DM can activate the Nrf2/HO-1 signaling pathway, thereby improving testicular OS and preventing the onset of OAT. Consistent with the results of Arab et al., the Nrf2/HO-1 signaling pathway activation could protect testicular tissue from oxidative damage and maintain good sperm quality [55]. Although this study shows that the improvement of OAT by DM may be related to the Nrf2/HO-1 signaling pathway, it has not been further verified. In future studies, we intend to use pathway suppressors and gene knockout methods to further explore the Nrf2/HO-1 signaling pathway.

## CONCLUSIONS

This study demonstrated the ability of moxibustion in preventing TGS-induced OAT occurrence. The effect of DM was better than that of ADM, and its mechanism appears to involve the regulation of Nrf2/HO-1 signaling pathway and the reduction of OS. Our findings also suggest a possible treatment for OAT that prevents male infertility.

## MATERIALS AND METHODS

### Animals

Male Sprague-Dawley rats, aged eight weeks and weighing between 250–280 g, were acquired from the Shanghai Xipuer-Bikai Lab Animal Co., Ltd., (license number: SCXK (HU) 2018-0006). The rats were maintained in a controlled environment with standard housing conditions, including an ambient temperature of 24–26°C, humidity levels ranging from 50–60%, and a 12-h dark/light round and provided with *ad libitum* access to water and food. The Animal Care Committee of Nanjing University of Chinese Medicine granted approval for the animal investigations, with reference number 202104A023.

### Grouping, model preparation, and intervention methods

A total of forty rats were allocated to four groups, namely CON, OAT, DM, and ADM randomly, employing a randomized digital table. Except for the CON group, the OAT model was established by gavage of 20 mg/kg TGS (Qianjin Xieli Pharmaceutical Co., Ltd., Z43020138, Hunan, China) once daily for 28 days. The CON group was administered an equivalent amount of a normal saline solution. Moxibustion intervention was conducted at the CV4, which is located 25 mm below the rat navel.

Following 1 h of gavage, the rats were administered an 2% sodium pentobarbital intraperitoneal injection (IP) (1.5 ml/kg; CAS: 57-33-0; Merck KGaA, Darmstadt, Germany) to mitigate any potential negative impacts associated with stress. Consistent with a previous method [56], we shaved the fur above the CV4 and transferred the rats to a customized moxibustion board. The moxibustion procedure involved the utilization of ignited moxa sticks, which had a length of 85 mm and diameter of 5.3 mm (Gosen Biological Co., Ltd., located in Hunan, China). The sticks were securely fastened to a tailored moxibustion board and were situated 1 cm superior to the targeted acupoint. The two different moxibustion groups received 10 mins of either DM or ADM for 28 consecutive days.

After treatment, the rats were anaesthetized with IP of 2% sodium pentobarbital (2.5 mL/kg). Blood samples were taken from their abdominal aorta. The testicular organs (testes, caput epididymis, and cauda epididymis) were isolated; the cauda epididymis was used for the detection of quality and DFI of sperm, while the caput epididymis and half of the left testis were treated with 4% paraformaldehyde for IHC and hematoxylin and eosin (H&E) staining. The remaining testicular tissue was used for the OS-related index assessment.

### Sperm quality

The assessment of sperm quality included, sperm motility (PR, PR+NP), sperm viability, sperm concentration and sperm malformation rate.

### Sperm motility

Under a microscope (Nikon E100, Tokyo, Japan), spermatozoa were selected from at least five different fields (400×) and sperm motility was quantified by visual assessment. Following World Health Organization directions [57], sperm motility was categorized into: (1) spermatozoa with PR motility, (2) spermatozoa with NP motility, and (3) non-motile spermatozoa. Sperm motility was then calculated.

### Sperm viability

Sperm viability was visualized using Sperm Living Stain solution (Leagene Biological Co., Ltd., DA0184, Beijing, China) as previously described [58]. To analyze the shape of sperm, a 10-μL suspension of sperm was applied onto a pristine glass slide to create a smear, which was subsequently allowed to dry in the air. The number of dead sperm (red or dark pink heads) and live sperm (white or pale pink heads) was recorded (Supplementary Figure 1A), and data were presented as a percentage.

### Sperm concentration

The cauda epididymides were immersed in a test pipe with 4 ml of preheated typical saline at 37°C and subsequently sectioned into smaller pieces to facilitate the release of semen. The semen sample was subjected to gentle agitation and subsequently incubated at 37°C in a water bath maintained for a duration of 5 min. Sperm suspensions from the rats were then extracted, and the spermatozoa were counted using an improved Neubauer hemocytometer (Qiu Jing, Shanghai, China) to determine the quantity of spermatozoa present in 1 ml of semen.

### Sperm malformation rate

The analysis of sperm morphology was performed in accordance with previously established protocols [59]. The sperm sample was utilized to prepare a seminal smear on a pristine glass slide. The smear was subsequently fixed in 4% paraformaldehyde and stained using 2% eosin obtained from Phygene Biological Co., Ltd., (PH0720, Fuzhou, China). The quantification of sperm malformation was conducted employing a light microscope, and subsequently, the rate of malformation of sperm was determined. The deformations of sperm (Supplementary Figure 1B) are primarily observed in the head and tail regions.

These malformations can be classified into various types, such as the absence of hook, banana-shaped, amorphous, fat head, tail fold, and double head. Spermatozoa that lacked a tail exhibited overlapping heads or demonstrated complete overlap with other spermatozoa were excluded from the totals.

### Sperm chromatin structure assay

The stability of sperm chromatin construction was assessed through the utilization of the sperm chromatin structure test, following previous methods [60, 61] employing an acridine orange (AO) fluorescence staining kit (Genmed Scientifics Inc., HL14018.1v. A, Boston, MA, USA). The sperm specimens underwent a cleaning process and were subsequently diluted with a clean buffer solution. The concentration of the sperm was then modified to 1 × 10^6^ sperm/ml. Subsequently, the DNA at the locations of strand breaks was denatured by exposing the specimens to an acidic solution with a pH of 1.20 for 30 s. Following which sperm samples were combined with 0.2 ml of AO staining solution and incubated for 5 min at ambient temperature. The samples were then re-suspended to facilitate flow cytometric analysis (Beckman, Pasadena, CA, USA). A sample size of 10,000 cells was subjected to analysis at a flow rate ranging from 200 to 250 cells per second. The fragmented sperm DNA, which exhibits a red fluorescence, is bound by AO, whereas the double-stranded DNA displays a green fluorescence. The DFI was detected as the quotient of red fluorescence to total fluorescence and provided the proportion of spermatozoa with fragmented DNA.

### H&E staining

The caput epididymis and testis were treated in 4% paraformaldehyde and placed at 4°C for 48 h, dehydrated in solutions of elevated ethanol levels, cleared with xylene, immersed in paraffin, and segmented into 6-μm segments. H&E staining was conducted according to routine protocols, and a light microscope was utilized to evaluate the pathological changes (U-ND6-2 Fluorescence Multifunctional Microscope, Olympus, Tokyo, Japan).

Representative indicators of seminiferous tubule and epididymal tubule components measured included the area and diameter of seminiferous tubules, the epididymal tubule diameter, the seminiferous epithelium height, and the height of the epididymal epithelial. All tubules were evaluated employing Version 1.8.0 of the Image J program (National Institutes of Health, Bethesda, MD, USA). LSJ performed the measurement procedure according to previous methods [62, 63]. The subsequent particulars were documented (Supplementary Figure 2A, 2B): (1) The diameter of the seminiferous tubule and the distance between its luminal borders were measured. (2) The region extending from the base to the center of the lumen was documented as the seminiferous tubules area. (3) The measurement from the basal membrane to the luminal border was regarded as the seminiferous epithelium height, and four epithelial heights that were perpendicular to each other were computed. The mean value was determined as the seminiferous epithelium height of this particular tubule. The epididymal tubule diameter and epithelial height calculation methods were the same as those for the seminiferous tubules.

### Serum reproductive hormone test

The levels of serum FSH, T, LH, and E_2_ were quantified employing commercially available enzyme-linked immunosorbent assay kits (E-EL-R0391c, E-EL-0155c, E-EL-R0026c, E-EL-0152c, Elabscience, Wuhan, China), following the directions provided by the manufacturer. The Microplate Reader (ELX800, Bio Tek, Winooski, VT, USA) was utilized to measure the optical densities (OD) at 450 nm.

### OS and anti-oxidant status assessment

The testicular tissues were homogenized to a 10% tissue homogenate employing a homogenizer and subsequently centrifuged at 2,500 rpm for 10 min to obtain the supernatant. The protein level was also detected using spectrophotometer quantification (UV-3100, MAPADA, Shanghai, China). OS and anti-oxidant status in testicular tissues were assessed quantitatively employing a commercial kit (Jiancheng Bioengineering Institute, A015-1-2, A001-1-1, A003-1-1, Nanjing, China) to measure the MDA, T-SOD, and T-AOC. A commercial enzyme-linked immunosorbent assay kit (E-EL-0028c, Elabscience, Wuhan, China) was used to quantitatively assess 8-OHdG.

### IHC staining

All testicular tissue slides were de-waxed and rehydrated. After performing antigen retrieval employing citrate buffer, the activity of endogenous peroxidase was inhibited by utilizing 3% hydrogen peroxide, and non-specific labeling was prevented by employing 5% bovine serum albumin.

Subsequent to the preparation of testicular segments, they incubated overnight at a temperature of 4°C with primary antibodies against Nrf2 (1:100 dilution cat. No. ab31163, Abcam, Cambridgeshire, UK) and HO-1 (1:200 dilution cat. No. ab13243, Abcam). On the subsequent day, the slides underwent a wash with phosphate-buffered saline, followed by an incubation period of thirty minutes at 37°C with a horseradish peroxidase-conjugated goat anti-rabbit IgG (SA1022, Boster, Wuhan, China). Diaminobenzidine (AR1022, Boster, Wuhan, China) was used to visualize immunoreactivity, followed by hematoxylin for nuclear counterstaining. The cells with positive staining were visualized using a 400× microscope (U-ND6-2 Fluorescence Multifunctional Microscope, Olympus, Tokyo, Japan). The quantification of the stained cells was conducted employing the Image J program, and the outcomes were expressed as the mean absorbance value.

### qRT-PCR

The RNA extraction process from rat testicular tissue was conducted employing a kit for RNA extraction. The concentration and purity of the obtained RNA were assessed employing the NanoDrop 1000 (Thermo Fisher Scientific, Rockford, IL, USA). The cDNA was produced using the HiScript II 1st Strand cDNA Synthesis Kit (R211-01, Vazyme, Nanjing, China) following the manufacturer’s instructions, followed by PCR amplification of the synthesized cDNA. The qRT-PCR reaction was executed in the following manner: initial pre-denaturation at 95°C for 5 min, succeeded by 40 denaturation cycles for 10 s at 95°C, annealing for 20 s at 60°C, and extension for 20 s at 72°C. Table 1 lists the specific primers utilized for detecting the target gene sequences. The relative expressions of the target genes have been identified employing the Ct (2^−ΔΔCt^) technique. Normalization of the fold-change in gene expression was performed with respect to an internal control gene, namely glyceraldehyde-3-phosphate dehydrogenase (GAPDH).

**Table 1:** Primer sequences for qRT-PCR.

**Gene**		**Primer sequence (5′–3′)**	**Product length**
Nrf2	Forward	GCCTTCCTCTGCTGCCATTAGTC	110
	Reverse	TGCCTTCAGTGTGCTTCTGGTTG	
HO-1	Forward	CAGGTGTCCAGGGAAGGCTTTAAG	96
	Reverse	TGGGTTCTGCTTGTTTCGCTCTATC	
GAPDH	Forward	TGCCACTCAGAAGACTGTGG	129
	Reverse	TTCAGCTCTGGGATGACCT	

### Western blotting

The testicular tissue was subjected to lysis employing a buffer solution that contained protease and phosphatase suppressors. The tissue was ground employing a grinder and subsequently lysed on ice for 15 min. Following centrifugation at a temperature of 4°C, the resultant supernatant was gathered. The quantification of total protein was performed utilizing a bicinchoninic acid assay kit (BL521A, Biosharp, Hefei, China). Subsequently, the specimen was blended with 5× sodium dodecyl sulfate-polyacrylamide gel electrophoresis sample-loading buffer (20315ES20, Yeasen, Shanghai, China) and subjected to denaturation at 100°C for 10 min. A 25-μg protein sample underwent separation via 8% sodium dodecyl sulfate-polyacrylamide gel electrophoresis for the purpose of detecting a specific protein. The target protein was subsequently transferred onto a polyvinylidene fluoride membrane (IEVH00005, Millipore, Boston, MA, USA) and subjected to a blocking step with 5% skimmed milk at 20–25°C for 2 h. Subsequently, the polyvinylidene fluoride membrane was treated with the corresponding primary antibodies: Nrf2 (1:1,000 dilution cat. No. WL02135, Wanleibio, Shenyang, China), HO-1 (1:2,000 dilution cat. No. ab13243, Abcam, Cambridgeshire, UK), and β-actin (1:5,000 dilution cat. No. 81115-1-RR, Proteintech, Chicago, IL, USA) at 4°C overnight. The membrane was then incubated for 1 h at 20–25°C with a secondary antibody (1:10,000 dilution cat. No. abs20145, Absin, Shanghai, China) and visualized using an enhanced chemiluminescence reagent (P2200, NCM Biotech, Suzhou, China). The gray values were analyzed using ImageJ software to calculate the relative expression.

### Statistical analysis

The data that adhered to a normal distribution are represented as the mean ± standard deviation $\left(\bar{x}\pm s\right)$; one-way ANOVA was employed for comparing multiple groups. In cases where homogeneity of variance was observed, the least significant difference technique was used to analyze multiple comparisons. Alternatively, Tamhane’s method was employed to analyze such comparisons if homogeneity of variance was not present in the data. The sperm quality and DFI were examined using the chi-square test. The statistical program SPSS (Version 26.0, IBM, Armonk, NY, USA) was utilized for conducting all statistical analyses. The level of statistical significance was set at *P* < 0.05.

### Data availability statement

The original contribution of this article has been fully reflected in the article/supplementary materials. For further inquiries, please contact the corresponding author.

## Supplementary Materials

Supplementary Figures
