# Supplementary material for: Moxibustion prevents tripterygium glycoside-induced oligoasthenoteratozoospermia in rats via reduced oxidative stress and modulation of the Nrf2/HO-1 signaling pathway
Source: Aging (Albany NY). 2024 Jan 25;16(3):2141–60. doi: 10.18632/aging.205475 (PMC10911353; doi:10.18632/aging.205475)
Supplement: Supplementary Figures [file aging-16-205475-s001.pdf]

SUPPLEMENTARY FIGURES

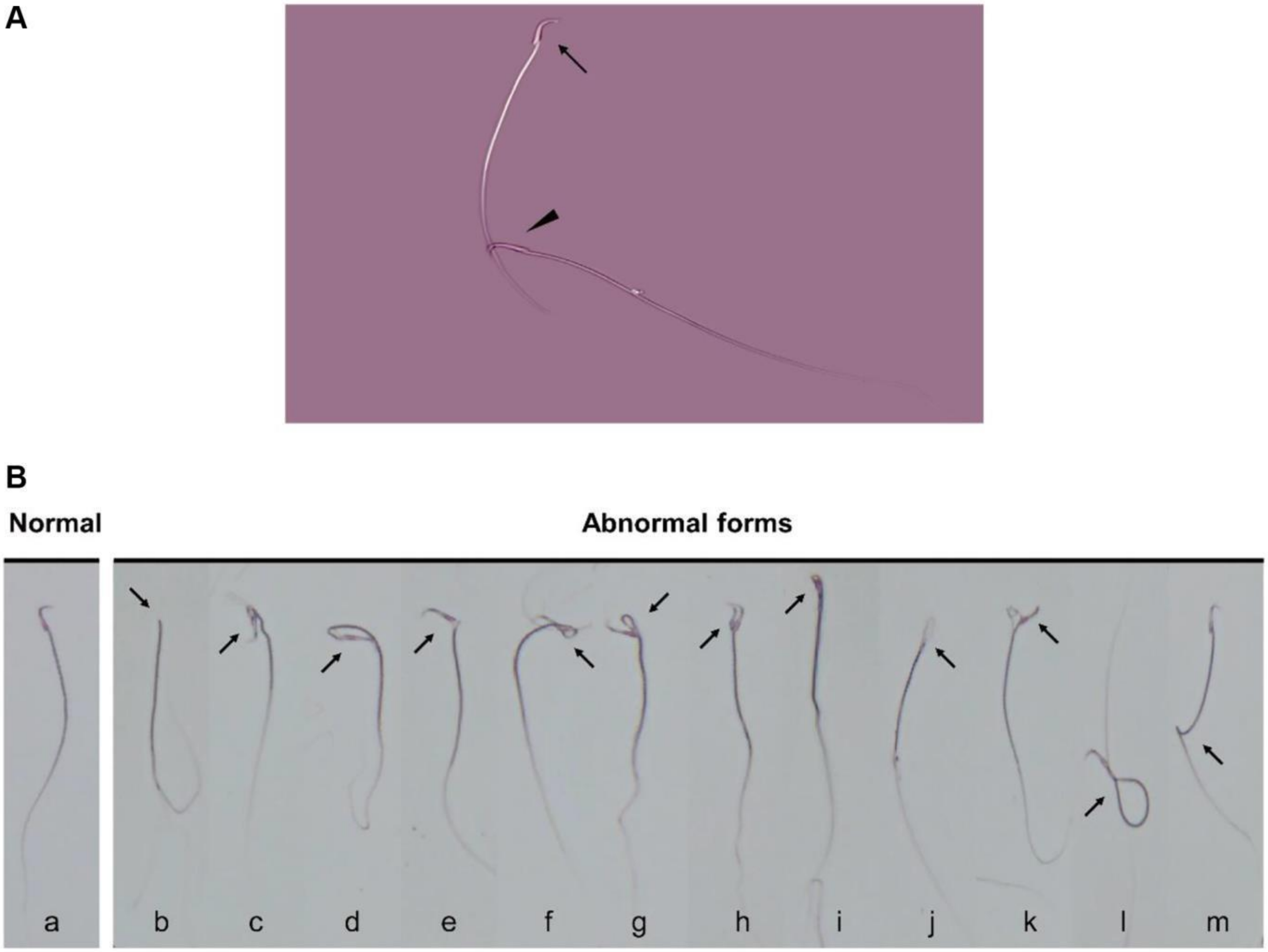

Supplementary Figure 1. Morphological manifestations of sperm survival and sperm malformation. (**A**) Live sperms manifested white or pale pink heads and dead sperms showed red or dark pink heads. Black arrow: live sperm; black triangle: dead sperm. (**B**) Normal sperm and abnormal sperm morphology, (**a**) Normal sperm with a sickle-shaped head and a long tail; (**b**) Disappearance of sperm heads; (**c**, **d**) Sperm head folded back pointing to tail tip; (**e**, **f**) Sperm head folded forward pointing to the tail; (**g**, **h**) Double head; (**I**, **j**) Fat head; (**k**) sickle-shaped head of sperm becomes straight; (**l**, **m**); Fold tail. Arrows show the abnormal parts of malformed sperms.

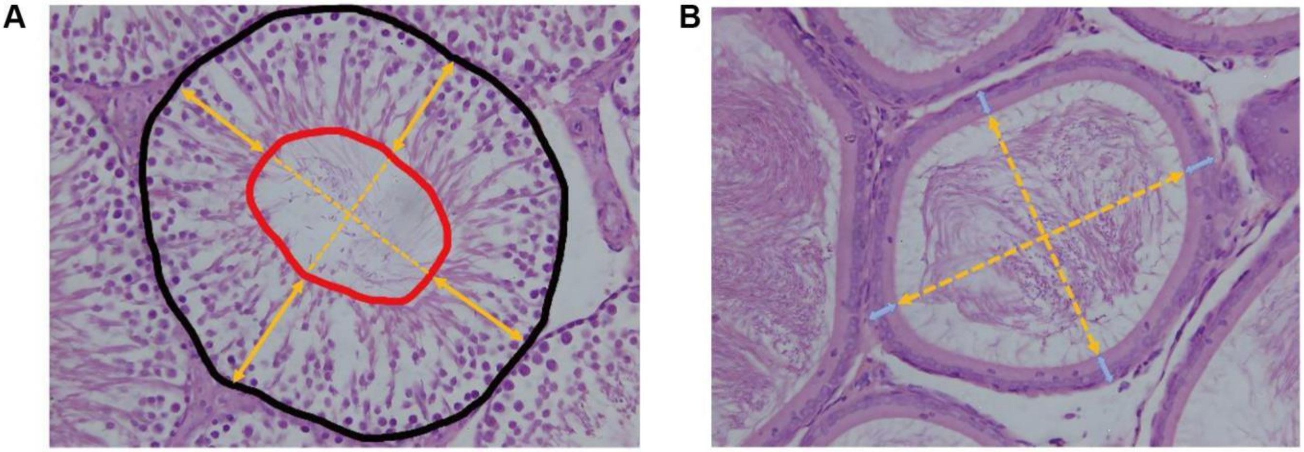

Supplementary Figure 2. Measurement of seminiferous tubules and epididymal tubules components. (**A**) The area in red line defines the area of a seminiferous tubule. The average length of two yellow vertical lines passing through the center represented the diameter of seminiferous tubules. The average length of the four yellow arrows represented the height of seminiferous epithelium. (**B**) The average length of the four yellow arrows represented the height of epididymal epithelial. The average length of two yellow vertical lines passing through the center represented the diameter of epididymal tubules.
